# Cancer-associated fibroblasts-derived extracellular vesicles carrying lncRNA SNHG3 facilitate colorectal cancer cell proliferation via the miR-34b-5p/HuR/HOXC6 axis

**DOI:** 10.1038/s41420-022-01116-z

**Published:** 2022-08-03

**Authors:** Jiangning Zhao, Huanrong Lin, Kunsong Huang, Shen Li

**Affiliations:** 1 Gastrointestinal Peritoneal Cancer Surgery, The Fourth Clinical Medical School of Guangzhou University of Chinese Medicine, Shenzhen Traditional Chinese Medicine Hospital, Shenzhen, 518033 Guangdong China; 2 Department of General Surgery, Guangzhou Overseas Chinese Hospital, The First Affiliated Hospital of Jinan University, Guangzhou, 510630 Guangdong China

**Keywords:** Colon cancer, Rectal cancer

## Abstract

Cancer-associated fibroblasts (CAFs)-derived extracellular vesicles (EVs) can mediate tumorigenesis. Long noncoding RNA (LncRNA) SNHG3 is implicated in colorectal cancer (CRC) progression. The current study sought to clarify the role of CAFs-EVs carrying SNHG3 in CRC cell proliferation. Firstly, CAFs and normal fibroblasts (NFs) were cultured and identified, followed by isolation and characterization of CAFs-EVs and NFs-EVs. CRC cells were cultured with CAFs-EVs or CAFs-EVs overexpressing SNHG3. The effects of SNHG3 on CRC cell proliferation was evaluated using CCK-8, colony formation, and EdU staining assays. The binding relationships among SNHG3, miR-34b-5p, and HuR were validated, in addition to analyzing the binding between HuR and HOXC6. Lastly, xenograft tumor model was established to verify the role of CAFs-EVs carrying SNHG3 in vivo. SNHG3 was highly expressed in CRC cells and CAFs-EVs, whereas CAFs-EVs facilitated CRC cell proliferation. Mechanically, CAFs-EVs carried SNHG3 into CRC cells to upregulate HuR expression by competitively binding to miR-34b-5p, promote the binding of HuR and HOXC6, and enhance HOXC6 transcription. miR-34b-5p over-expression or HOXC6 silencing annulled the effect of CAFs-EVs. SNHG3 carried by CAFs-EVs facilitated CRC proliferation *via* the miR-34b-5p/HuR/HOXC6 axis in vivo. Collectively, our findings indicated that CAFs-EVs carried SNHG3 into CRC cells to upregulate HuR expression by sponging miR-34b-5p and finally enhance HOXC6 transcription, thereby facilitating CRC cell proliferation.

## Facts

1. CAFs-EVs promote the proliferation of CRC cells.
2. CAFs-EVs carry SNHG3 into CRC cells.
3. SNHG3 upregulates HuR by sponging miR-34b-5p and enhances HOXC6 transcription.
4. miR-34b-5p overexpression or HOXC6 silencing reduces the effect of CAFs-EVs.
5. SNHG3 carried by CAFs-EVs facilitates CRC proliferation via miR-34b-5p/HuR/HOXC6.

## Introduction

Colorectal cancer (CRC) is precipitated by the gradual accumulation of genetic and epigenetic alternations, resulting in the deterioration of normal colonic mucosa to invasive cancer [1]. Numerous risk factors such as age, genetic and environmental factors contribute to CRC development, while emerging studies have shown that inflammatory bowel disease, obesity, sedentary lifestyle, and coronary artery diseases also contribute to CRC initiation and development [2]. Surgery prevails as the gold-standard treatment for early CRC cases, however, a large majority of CRC patients usually get diagnosed at advanced stages and sometimes present with distant metastases, leading to unsatisfactory outcomes and dismal survival [3]. Hence, it is prudent to further clarify the exact molecular mechanism behind CRC initiation and progression to provide novel insight for potential diagnostic or therapeutic strategies against CRC.

The last few decades have seen great advancements in the investigation of CRC; the scientific community no longer merely focuses on cancer cells but has also focused their efforts on the role of the tumor microenvironment (TME) in promoting CRC progression [4]. Among the stromal cells present in the TME, cancer-associated fibroblasts (CAFs) represent the most abundant and crucial component in tumor mesenchyme, and further known to exert strong tumor-modulating effects [5, 6]. Meanwhile, there is evidence to suggest that the interaction between cancer cells and CAFs in TME augments the carcinogenesis of CRC, which underscores the potential of the CAF profile as a biomarker of CRC progression [4]. Moreover, the accumulation of stromal cells in TME, especially CAFs around adenomatous colorectal polyps and primary tumor sites, is associated with unfavorable prognoses and high recurrence rates of CRC [7]. Accordingly, targeting CAFs in TME can be regarded as a promising therapeutic avenue to realize positive outcomes in CRC patients.

Furthermore, CAFs carry out their role of maintaining tumor characteristics by means of secreting soluble paracrine signals and releasing extracellular vesicles (EVs) [8]. Meanwhile, the aforementioned EVs, as a group of membrane-enclosed vesicles, serve as the crucial medium of intercellular communication in TME and participate in a plethora of processes associated with tumor progression, including proliferation, angiogenesis, and metastasis [9]. Additionally, EVs secreted by CAFs have been previously indicated to confer radioresistance in CRC cells and expedite tumor growth in irradiated mice, leading to poorer clinical outcomes [10]. Besides, CAFs-EVs also possess the ability to enhance epithelial-mesenchymal transition (EMT) and facilitate metastasis of CRC cells by increasing cell stemness [11]. Moreover, the capacity of CAFs-EVs to modulate cancer progression largely depends on the content transfer of their cargoes including proteins, mRNAs, and noncoding RNAs, to cancer cells [11]. On a separate note, long noncoding RNAs (lncRNAs), represent a class of transcripts with over 200 nucleotides that lack apparent protein-coding capacity [12]. As critical contributors to the epigenetic mechanism, dysregulation of lncRNAs leads to CRC carcinogenesis and malignant transformation [13]. One such lncRNA, namely small nucleolar RNA host gene 3 (SNHG3), is aberrantly up-regulated in CRC patients, such that SNHG3 up-regulation is associated with advanced clinical stage, distant metastasis, and dismal overall survival [3, 14]. It is also noteworthy that, a prior study has illustrated that SNHG3 is carried by CAFs-EVs to accelerate breast cancer cell growth [15]. Nevertheless, whether CAFs-EVs can play a role in CRC cell proliferation by carrying SNHG3 remains elusive. Herein, the current study set out to investigate the potential effects of CAFs-EVs carrying SNHG3 on CRC cell proliferation, hoping to confer novel insights for the management of CRC.

## Results

### Isolation and characterization of NFs, CAFs, and their EVs

A number of studies have explored the role of EVs in human cancers [16, 17], while the role of CAFs-EVs carrying lncRNAs in CRC remains elusive. Accordingly, we isolated and cultured CAFs in CRC tissues and NFs in normal tissues. Microscopic observation revealed that NFs presented flat stellate with multiple cytoplasmic processes, basically the same cell size, and ordered arrangement, while CAFs were spindle or long spindle-shaped, with inconsistent cell size, and disorderly arranged (Supplementary Fig. 1A). In addition, we detected the expression patterns of cell-specific markers, and the results of immunocytochemical assay exhibited that vimentin in NFs was positively expressed, and keratin and α-SMA were negatively expressed, whereas in CAFs, vimentin and α-SMA were positively expressed, and keratin was negatively expressed (Supplementary Fig. 1B). Meanwhile, RT-qPCR assay also illustrated that α-SMA and FAP mRNA expression levels were notably higher in CAFs compared to those in NFs, while there were no differences in vimentin mRNA expressions between NFs and CAFs (Supplementary Fig. 1C), which validated that we isolated typical NFs and CAFs. Subsequently, we isolated the EVs from NFs and CAFs, and TEM illustrated that, the particles were typical cup-shaped and the particle size ranged from 50 to 150 nm (Supplementary Fig. 1D). Moreover, NTA demonstrated that the average diameter of NFs-EVs was 124.9 ± 11.6 nm and the concentration was 9.0 × 10^6^ particles/mL, while the average diameter of CAFs-EVs was 138.7 ± 18.2 nm and the concentration was 1.0 × 10^7^ particles/mL (Supplementary Fig. 1E). Besides, the results of Western blot assay illustrated that CD9 and CD63 were positively expressed in NFs-EVs and CAFs-EVs, while calnexin was negatively expressed (Supplementary Fig. 1F and Supplementary Fig. 3). Together, these findings indicated that the extracted EVs met the requirements for subsequent experimentation.

### CAFs-EVs facilitated CRC cell proliferation

**Fig. 1:**
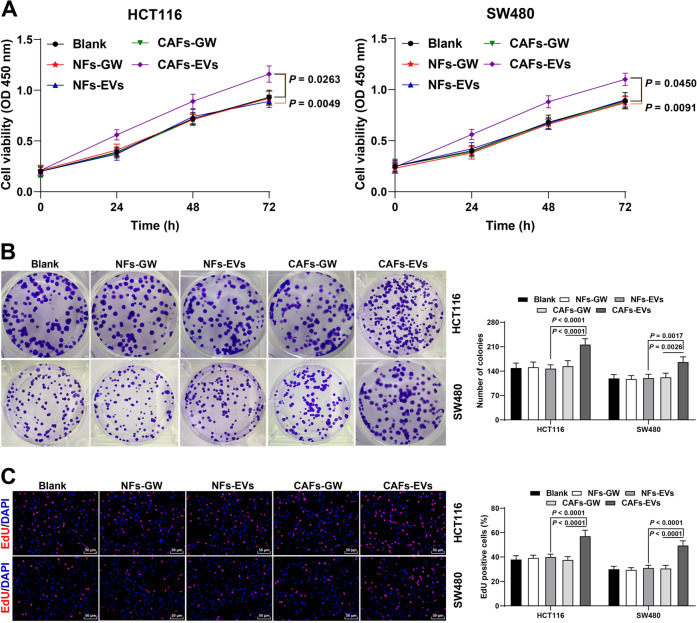
CAFs-EVs promoted the proliferation of CRC cells. CRC cells were treated with NFs-EVs and CAFs-EVs, with NFs-GW and CAFs-GW treatment as negative control. **A**–**C** The cell proliferation ability was measured using CCK-8 assay (**A**), colony formation assay (**B**), and EdU staining (**C**). The cell experiment was repeated 3 times independently. Data are presented as mean ± standard deviation. Data in panel A were analyzed using two-way ANOVA, and data in **B**, **C** were analyzed using one-way ANOVA, followed by Tukey’s multiple comparisons test.

Subsequently, we treated CRC cells with EVs to investigate the effect of CAFs-EVs on CRC cell proliferation. The results of CCK-8 and colony formation assays showed that NFs-EVs did not alter CRC cell proliferation ability, whereas CAFs-EVs notably facilitated CRC cell proliferation. There was a reduction in the promoting effect of CAFs-EVs on CRC cell proliferation after the addition of GW4869 to depress the generation of CAFs-EVs (CAFs-GW) (Fig. 1A, B). In addition, the results of EdU staining further validated the above findings (Fig. 1C). Briefly, CAFs-EVs promoted the proliferation of CRC cells.

### CAFs-EVs carried SNHG3 into CRC cells

**Fig. 2:**
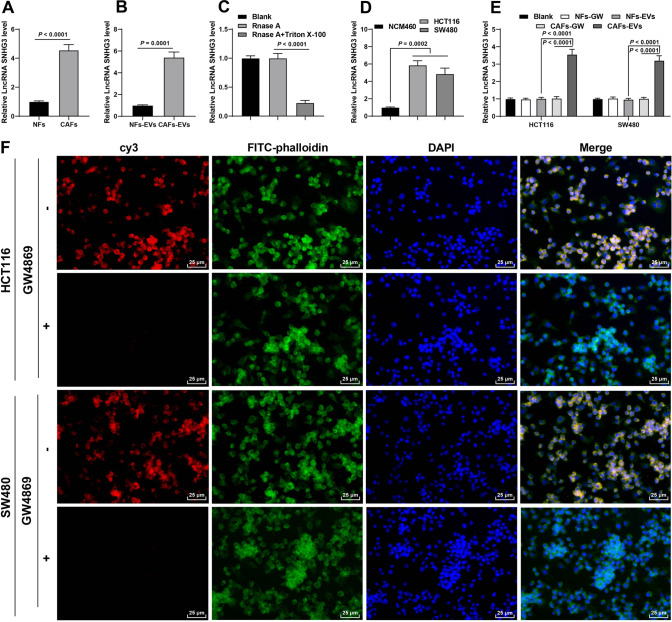
CAFs-EVs carried SNHG3 into CRC cells. **A** SNHG3 expression in NFs and CAFs was determined using RT-qPCR. **B** SNHG3 expression in NFs-EVs and CAFs-EVs was determined using RT-qPCR. **C** After Rnase A treatment or the combined treatment of Rnase A and Triton-X-100, SNHG3 expression in CAFs was determined using RT-qPCR. **D**, **E** SNHG3 expression in NCM460 and CRC cells was determined using RT-qPCR. **F** The delivery of cy3-labeled SNHG3 was observed under the confocal microscope. The cell experiment was repeated three times independently. Data are presented as mean ± standard deviation. Data in **A**, **B** were analyzed using *t-*test. Data in **C**–**E** were analyzed using one-way ANOVA, followed by Tukey’s multiple comparisons test.

Existing evidence suggests that SNHG3 can be encapsulated by CAFs-EVs [15], while SNHG3 is over-expressed in CRC [3, 14]. We further speculated that CAFs-EVs carried SNHG3 to influence the proliferation of CRC cells. It was found that SNHG3 expression levels in CAFs and CAFs-EVs were notably higher than those in NFs and NFs-EVs (Fig. 2A, B). Meanwhile, RNase A treatment did not affect the SNHG3 expression in CAFs, but RNase A treatment combined with Triton-X-100 resulted in significantly decreased SNHG3 expression levels (Fig. 2C), suggesting that SNHG3 was encapsulated in CAFs-EVs. Moreover, SNHG3 expression levels were elevated in CRC cells, while NFs-EVs treatment did not affect the SNHG3 expression, but CAFs-EVs treatment significantly elevated the SNHG3 expression levels (Fig. 2D, E). To further explore whether CAFs-EVs could deliver SNHG3 into CRC cells, we transfected cy3-labeled SNHG3 into CAFs, and then co-cultured with FITC-phalloidin-labeled CRC cells. Microscopic observation revealed that, there was a large amount of red fluorescence in FITC-labeled CRC cells, but no red fluorescence was found in CRC cells after the addition of GW4869 in the above system (Fig. 2F). Altogether, these findings indicated that CAFs-EVs transferred SNHG3 into CRC cells to upregulate SNHG3 expression in CRC cells.

### CAFs-EVs carrying SNHG3 enhanced CRC cell proliferation

**Fig. 3:**
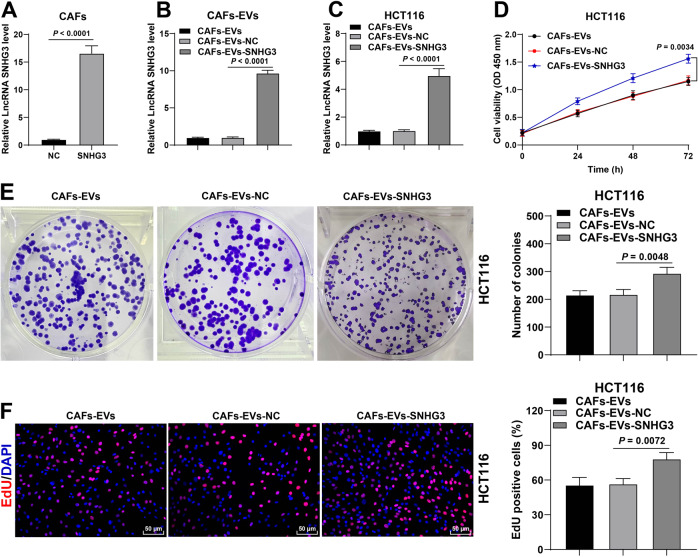
CAFs-EVs carrying SNHG3 enhanced the proliferation of CRC cells. CAFs were infected with lentivirus overexpression vector of SNHG3. **A** SNHG3 expression in CAFs was determined using RT-qPCR. EVs were isolated from CAFs overexpressing SNHG3 (CAFs-EVs-SNHG3) to treat HCT116 cells. **B** SNHG3 expression in CAFs-EVs was determined using RT-qPCR. **C** SNHG3 expression in HCT116 cells was determined using RT-qPCR. **D**–**F** The cell proliferation ability was measured using CCK-8 assay (**D**), colony formation assay (**E**), and EdU staining (**F**). The cell experiment was repeated three times independently. Data are presented as mean ± standard deviation. Data in **A** were analyzed using *t*-test. Data in **B**, **C**/**E**, **F** were analyzed using one-way ANOVA, and data in panel D were analyzed using two-way ANOVA, followed by Tukey’s multiple comparisons test.

To further verify the function of SNHG3 in the regulation of CRC cell proliferation by CAFs-EVs, we transfected CAFs with lentivirus over-expression vector of SNHG3 and the results of RT-qPCR verified the transfection efficiency (Fig. 3A). Subsequently, EVs were isolated from CAFs overexpressing SNHG3 (CAFs-EVs-SNHG3), and SNHG3 expression levels were found to be significantly elevated in CAFs-EVs-SNHG3 (Fig. 3B). Meanwhile, HCT116 cells were treated with CAFs-EVs-SNHG3 and SNHG3 expression in HCT116 cells was increased (Fig. 3C). Following over-expression of SNHG3 in HCT116 cells, there was a significant enhancement in the proliferation ability of cells (Fig. 3D, E). The results of EdU staining also illustrated that the proliferation of HCT116 cells was increased following CAFs-EVs-SNHG3 treatment (Fig. 3F). Collectively, these findings suggested that CAFs-EVs carried SNHG3 into CRC cells and enhanced the proliferation of CRC cells.

### SNHG3 suppressed miR-34b-5p expression

**Fig. 4:**
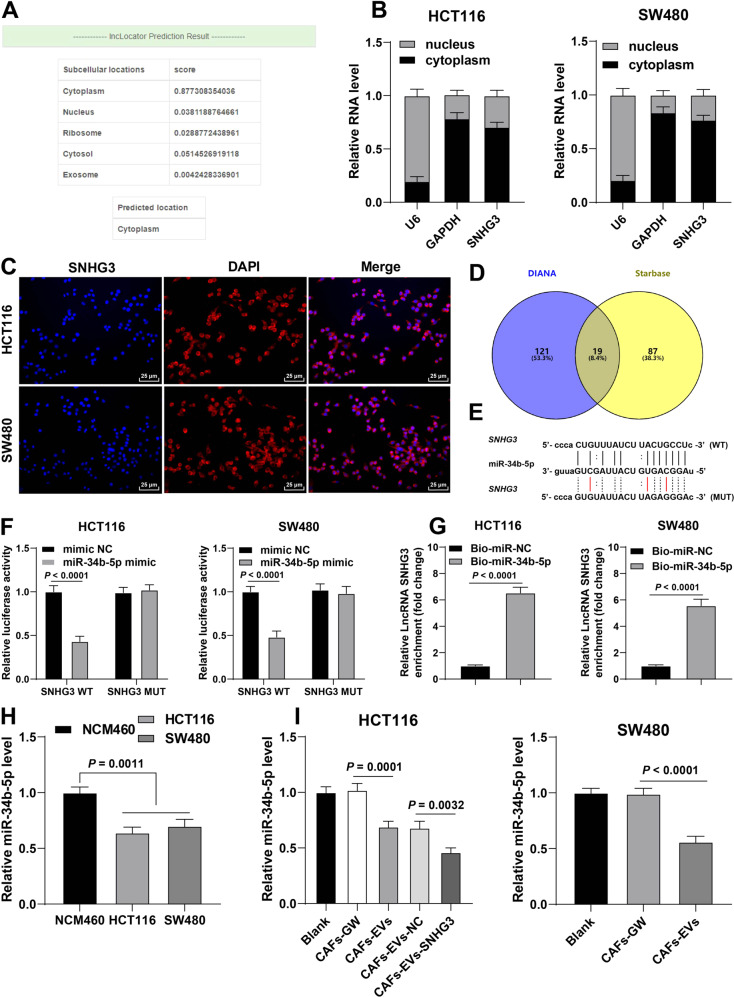
SNHG3 inhibited miR-34b-5p expression. **A** The subcellular localization of SNHG3 through the lnclocator software. **B**, **C** The location of SNHG3 in CRC cells was verified using nuclear/cytosol fractionation assay and RNA FISH. **D** The downstream miRNAs of SNHG3 were predicted through DIANA and Starbase databases. **E** The binding site of SNHG3 and miR-34b-5p in the Starbase database. **F**, **G** The binding relationship between SNHG3 and miR-34b-5p was verified using dual-luciferase assay and RNA pull-down. **H**, **I** miR-34b-5p expression in NCM460 and CRC cells was determined using RT-qPCR. The cell experiment was repeated three times independently. Data are presented as mean ± standard deviation. Data in **G** were analyzed using *t*-test. Data in **H**, **I** were analyzed using one-way ANOVA, and data in **F** were analyzed using two-way ANOVA, followed by Tukey’s multiple comparisons test.

Thereafter, we focused our efforts on the downstream mechanism of SNHG3, and predicted the subcellular localization of SNHG3 through the lnclocator software, which revealed SNHG3 was primarily localized in the cytoplasm (Fig. 4A). Moreover, the results of nuclear/cytosol fractionation assay and RNA FISH validated the location of SNHG3 in the cytoplasm of CRC cells (Fig. 4B, C), suggesting that SNHG3 played a role through the competitive endogenous RNA (ceRNA) mechanism [18]. In addition, we predicted the downstream miRNAs of SNHG3 (Fig. 4D), among which miR-34b-5p was poorly expressed in CRC [19, 20]. Based on the binding site of SNHG3 and miR-34b-5p in the Starbase database (Fig. 4E), we designed a dual-luciferase assay and confirmed the presence of a binding relationship between SNHG3 and miR-34b-5p in CRC cells (Fig. 4F). RNA pull-down assay further validated the binding relationship between SNHG3 and miR-34b-5p (Fig. 4G). miR-34b-5p was poorly expressed in CRC cells, and after CAFs-EVs and Besides, following CAFs-EVs-SNHG3 treatment, there was a significant reduction in miR-34b-5p expression levels (Fig. 4H–I). Briefly, these findings indicated that SNHG3 inhibited the expression of miR-34b-5p.

### miR-34b-5p overexpression suppressed the promoting effect of CAFs-EVs on CRC cell proliferation

**Fig. 5:**
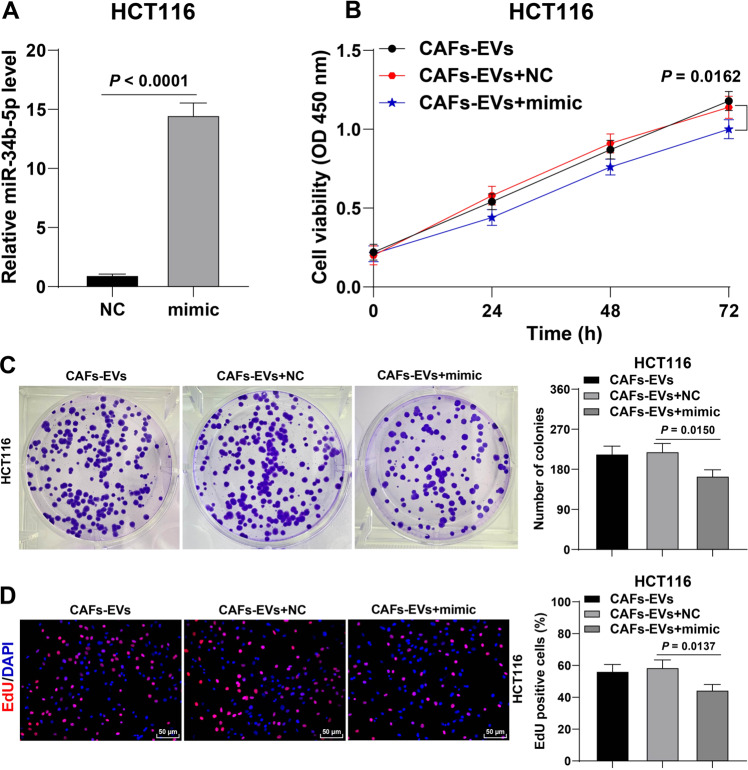
miR-34b-5p overexpression suppressed the promoting effect of CAFs-EVs on CRC cell proliferation. HCT116 cells were transfected with miR-34b-5p mimic, with mimic NC as negative control. Then HCT116 cells transfected with miR-34b-5p mimic were treated with CAFs-EVs. **A** miR-34b-5p expression in HCT116 cells was determined using RT-qPCR. **B**–**D** The cell proliferation ability was measured using CCK-8 assay (**B**), colony formation assay (**C**), and EdU staining (**D**). The cell experiment was repeated three times independently. Data are presented as mean ± standard deviation. Data in **A** were analyzed using *t-*test. Data in **C**, **D** were analyzed using one-way ANOVA, and data in **B** were analyzed using two-way ANOVA, followed by Tukey’s multiple comparisons test.

To further validate the role of miR-34b-5p in the regulation of CRC cell proliferation by CAFs-EVs, we transfected miR-34b-5p mimic into HCT116 cells and successfully up-regulated the miR-34b-5p expression in cells (Fig. 5A), followed by CAFs-EVs treatment. Following over-expression of miR-34b-5p, the promoting effect of CAFs-EVs on the proliferation of HCT116 cells was found to be dramatically inhibited (Fig. 5B–D). The aforementioned findings indicated that over-expression of miR-34b-5p suppressed the promotive effect of CAFs-EVs on CRC cell proliferation.

### miR-34b-5p targeted HuR expression, thereby inhibiting the binding of HuR to HOXC6 and reducing the stability of HOXC6 mRNA

**Fig. 6:**
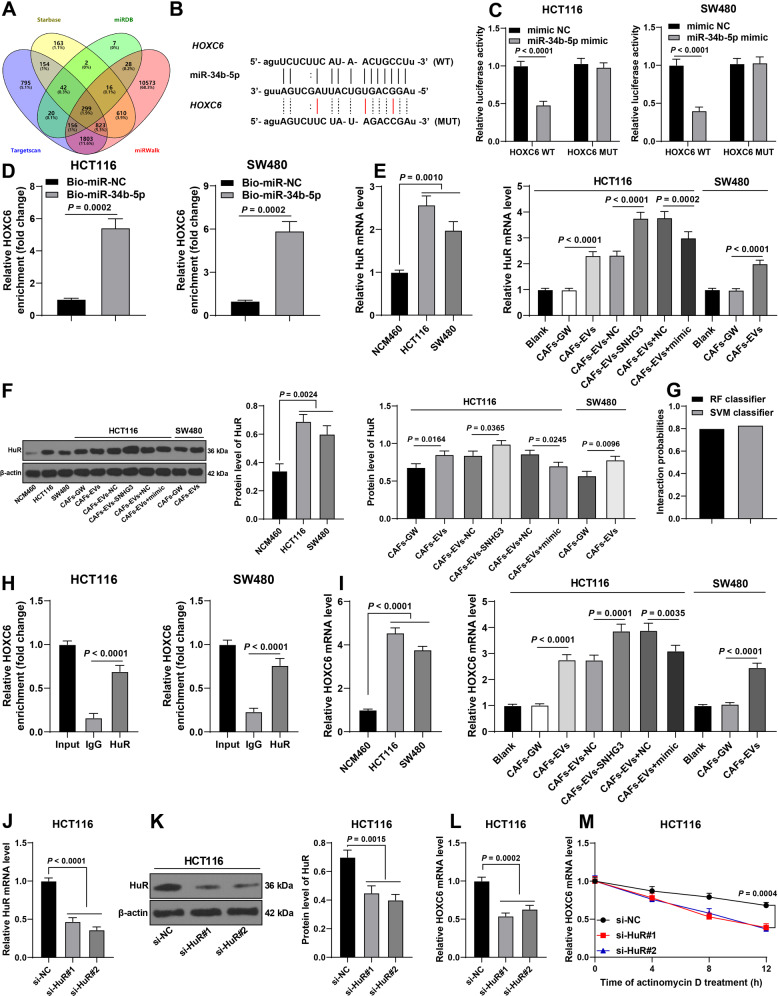
miR-34b-5p targeted HuR expression, thereby inhibiting the binding of HuR to HOXC6 and reducing the stability of HOXC6 mRNA. **A** The downstream genes of miR-34b-5p were predicted through Starbase, miRDB, Targetscan, and miRWalk databases. **B** The binding site of miR-34b-5p and HuR in the Starbase database. **C**, **D** The binding relationship between miR-34b-5p and HuR was verified using dual-luciferase assay and RNA pull-down assay. **E**, **F** HuR expression in NCM460 and CRC cells was determined using RT-qPCR and Western blotting. **G** The binding score of HuR and HOXC6 was predicted through the RPISeq database. **H** The binding of HuR and HOXC6 was analyzed using RIP assay. **I** HOXC6 mRNA expression in NCM460 and CRC cells was determined using RT-qPCR. HCT116 cells were transfected with HuR siRNA (si-HuR), with NC siRNA (si-NC) as negative control. **J**, **K** HuR expression in HCT116 cells was determined using RT-qPCR and Western blotting. **L**, **M** HOXC6 mRNA expression and half-life period in HCT116 cells were determined using RT-qPCR. The cell experiment was repeated three times independently. Data are presented as mean ± standard deviation. Data in **D**/**F** were analyzed using *t*-test. Data in **E**, **F**/**H**–**L** were analyzed using one-way ANOVA, and data in panels C/M were analyzed using two-way ANOVA, followed by Tukey’s multiple comparisons test.

Furthermore, we predicted the downstream genes of miR-34b-5p (Fig. 6A), among which embryonic lethal abnormal vision 1 (ELAVL1)/HuR expression was previously illustrated to be elevated in CRC [21]. Based on the binding site of miR-34b-5p and HuR in the Starbase database (Fig. 6B), we designed a dual-luciferase assay and confirmed the presence of a binding relationship between miR-34b-5p and HuR in CRC cells (Fig. 6C). The results of RNA pull-down assay further validated their binding relationship (Fig. 6D). Moreover, we found that HuR was highly expressed in CRC cells, while treatment with CAFs-EVs and CAFs-EVs-SNHG3 further enhanced the HuR expression, whereas miR-34b-5p over-expression reduced HuR expression levels (Fig. 6E, F and Supplementary Fig. 4). Moreover, prior studies have shown that HuR binds to the 3'UTR of target mRNA and promotes its stability [22, 23]. Meanwhile, HuR is also known to bind to HOXC6 and promotes its stability [24], whereas HOXC6 is highly expressed in CRC [25, 26]. Accordingly, we speculated whether HOXC6 was the downstream target gene of HuR. Results of RPISeq database analysis illustrated that HuR and HOXC6 exhibited a high binding score (Fig. 6G). Besides, RIP assay further validated the binding relationship between HuR and HOXC6 (Fig. 6H). We observed that HOXC6 was highly expressed in CRC cells, whereas treatment with CAFs-EVs and CAFs-EVs-SNHG3 further enhanced HOXC6 mRNA expression, while over-expression of miR-34b-5p decreased HOXC6 mRNA expression levels (Fig. 6I). To further validate the effect of HuR on HOXC6, we transfected HuR siRNA (si-HuR) into HCT116 cells. Following the successful down-regulation of HuR expression in cells (Fig. 6J, K and Supplementary Fig. 5), there was a reduction in HOXC6 mRNA expression levels (Fig. 6L) and its half-life was shortened (Fig. 6M). Altogether, these findings indicated that miR-34b-5p targeted HuR expression, thereby inhibiting the binding of HuR to HOXC6 and reducing the stability of HOXC6 mRNA.

### HOXC6 silencing depressed the promoting effect of CAFs-EVs on CRC cell proliferation

**Fig. 7:**
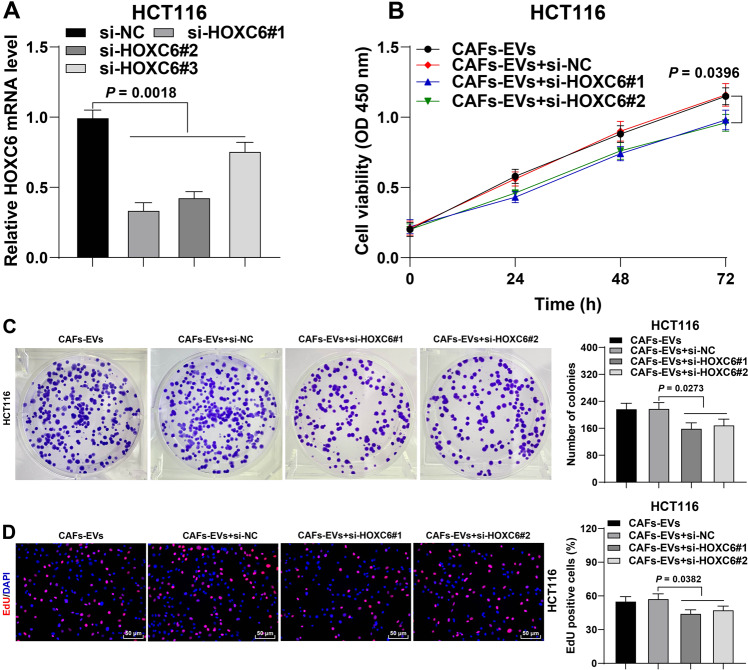
HOXC6 silencing depressed the promoting effect of CAFs-EVs on CRC cell proliferation. Three HOXC6 siRNAs (si-HOXC6) were transfected into HCT116 cells respectively, with NC siRNA (si-NC) as negative control. Then, si-HOXC6-transfected HCT116 cells were treated with CAFs-EVs. **A** HOXC6 mRNA expression in HCT116 cells was determined using RT-qPCR. **B**–**D** The cell proliferation ability was measured using CCK-8 assay (**B**), colony formation assay (**C**), and EdU staining (**D**). The cell experiment was repeated three times independently. Data are presented as mean ± standard deviation. Data in **A**/**C**, **D** were analyzed using one-way ANOVA, and data in **B** were analyzed using two-way ANOVA, followed by Tukey’s multiple comparisons test.

Furthermore, we verified the role of HOXC6 in the regulation of CRC cell proliferation by CAFs-EVs. Briefly, we transfected three HOXC6 siRNAs (si-HOXC6) into HCT116 cells, respectively and down-regulated HOXC6 mRNA expression in cells (Fig. 7A). Two siRNAs exhibiting good efficiency were selected to treat HCT116 cells with CAFs-EVs. Following HOXC6 silencing, there was a significant reduction in the promoting effect of CAFs-EVs on the proliferation of HCT116 cells (Fig. 7B–D). Briefly, these findings indicated silencing of HOXC6 depressed the promotive effect of CAFs-EVs on CRC cell proliferation.

### CAFs-EVs carrying SNHG3 enhanced CRC cell proliferation in vivo via the miR-34b-5p/HuR/HOXC6 axis

**Fig. 8:**
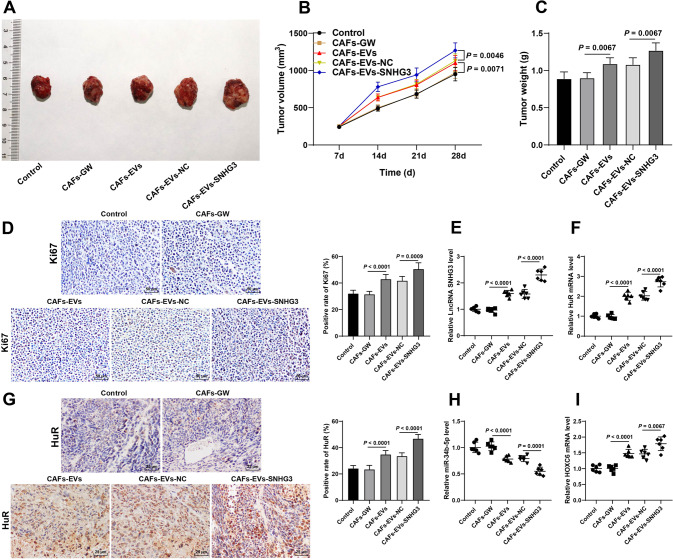
CAFs-EVs carrying SNHG3 enhanced CRC cell proliferation in vivo via the miR-34b-5p/HuR/HOXC6 axis. The murine xenograft model was established by CAFs-GW- or CAFs-EVs-treated HCT116 cells. **A** Representative images of xenograft tumor. **B** Tumor volume. **C** The tumor weight on the 28th day after euthanasia in nude mice. **D** The positive rate of ki67 in tumor tissues was detected using immunohistochemistry. **E**, **F** SNHG3 and HuR expressions in tumor tissues were determined using RT-qPCR. G: The positive rate of HuR in tumor tissues was detected using immunohistochemistry. **H, I** miR-34b-5p and HOXC6 expressions in tumor tissues were determined using RT-qPCR. *N* = 6 or *N* = 12. Data in **B**/**C**/**D**/**G** are presented as mean ± standard deviation. Data in **C**–**I** were analyzed using one-way ANOVA, and data in **B** were analyzed using two-way ANOVA, followed by Tukey’s multiple comparisons test.

Finally, we established a murine xenograft model with HCT116 cells to verify the mechanism of CAFs-EVs in vivo. It was found that, CAFs-EVs and CAFs-EVs-SNHG3 significantly facilitated tumor growth, as evidenced by increased tumor volume and weight (Fig. 8A–C), and elevated Ki67-positive rate (Fig. 8D). Following CAFs-EVs treatment, there was an increase in the expressions of SNHG3, HuR, and HOXC6, while the miR-34b-5p expression was decreased, and CAFs-EVs-SNHG3 treatment further augmented the above trends (Fig. 8E–I). Collectively, the aforementioned findings indicated that CAFs-EVs carrying SNHG3 enhanced CRC cell proliferation in vivo *via* the miR-34b-5p/HuR/HOXC6 axis.

## Discussion

CRC prevails as one of the most frequently diagnosed malignancies across the world, accompanied by increasing morbidity and mortality rates [13]. Meanwhile, the hard-done work of our peers has highlighted that lncRNA SNHG3 functions as a ceRNA to accelerate the progression of CRC [3]. It is also noteworthy that CAFs, the major constituents of tumor stroma, possess the ability to actively induce CRC tumorigenesis and progression [5], whereas EVs mediate the communication between cancer cells and CAFs in TME [15]. Accordingly, the current study sought to elucidate the potential effect of CAFs-EVs carrying SNHG3 on CRC cell proliferation, and our findings revealed that CAFs-EVs carrying SNHG3 enhanced CRC cell proliferation *via* the miR-34b-5p/HuR/HOXC6 axis (Supplementary Fig. 2).

CAFs-EVs are well-established to be capable of inducing aggressive CRC phenotypes [27], increasing CRC cell stemness [28], and also facilitating liver metastasis [29]. Herein, in our study, we isolated CAFs-EVs and NFs-EVs to treat CRC cells, and found that NFs-EVs did not alter CRC cell proliferation, whereas CAFs-EVs notably facilitated CRC cell proliferation. On the other hand, the addition of GW4869 to inhibit the generation of CAFs-EVs brought about a significant reduction in the promoting effect of CAFs-EVs on cell proliferation, which indicated that CAFs-EVs enhanced CRC cell proliferation. Meanwhile, the ability of CAFs-EVs to mediate tumor initiation and progression has been associated with the delivery of various molecular cargoes, such as lncRNAs [8]. For instance, a prior study has highlighted that CAFs-EVs facilitate breast cancer cell proliferation by means of transferring lncRNA SNHG3 [15]. Interestingly, SNHG3, a newly identified lncRNA, was previously documented to be highly expressed in CRC cells, and further associated with poor survival and dismal prognosis in CRC patients [3, 14]. At that conjecture, we speculated whether CAFs-EVs facilitated CRC cell proliferation by carrying SNHG3. Subsequent experimentation illustrated that SNHG3 expression levels were notably elevated in CAFs and CAFs-EVs. In addition, RNase A treatment did not affect SNHG3 expression in CAFs, whereas RNase A treatment combined with Triton-X-100 exerted a diminishing effect on SNHG3 expression levels, indicating that SNHG3 was encapsulated in CAFs-EVs. Consistently, our findings also exhibited the over-expression of SNHG3 in CRC cells, such that CAFs-EVs transferred SNHG3 into CRC cells to upregulate SNHG3 in CRC cells. Furthermore, we over-expressed SNHG3 in CAFs and isolated the formed EVs (CAFs-EVs-SNHG3). It was found that CAFs-EVs-SNHG3 treatment up-regulated SNHG3 expression levels, in addition to notably enhancing CRC cell proliferation. In accordance with our findings, a prior study indicated that SNHG3 over-expression expedites CRC cell proliferation, whereas silencing of SNHG3 impairs the malignant development of CRC [3]. Altogether, these findings and evidences exhibited that CAFs-EVs carried SNHG3 into CRC cells to enhance CRC cell proliferation.

Thereafter, we focused our efforts on exploring the downstream mechanism of SNHG3 in regulating CRC cell proliferation. LncRNAs are known to serve as ceRNAs by sponging microRNAs (miRNAs) by means of their miRNA response elements, thus ultimately interfering with the expression of their target mRNAs [30]. Interestingly, the aforementioned lncRNA/miRNA/mRNA network has been documented in numerous biological processes of CRC, such as liver metastasis, EMT, and chemotherapy/radiotherapy resistance [31]. Our findings further validated the location of SNHG3 in the cytoplasm of CRC cells, which suggested that SNHG3 played a role through the ceRNA mechanism. Meanwhile, miRNAs, as a class of small endogenous noncoding RNAs (18–25 nt in length), possess the ability of modulating gene expression at the post-transcriptional level [32]. One such miRNA, namely miR-34b-5p, was previously shown to function as a tumor suppressor in CRC [19, 20]. Dual-luciferase and RNA pull-down assays in our study confirmed the presence of a binding relationship between SNHG3 and miR-34b-5p in CRC cells. In addition, miR-34b-5p expression levels were found to be diminished in CRC cells, whereas treatment with CAFs-EVs or CAFs-EVs-SNHG3 further down-regulated miR-34b-5p expressions, underscoring that SNHG3 targeted miR-34b-5p. Additionally, we validated the function of miR-34b-5p in CRC cell proliferation. HCT116 cells were transfected with miR-34b-5p mimic and treated with CAFs-EVs. Notably, a prior study indicated that silencing miR-34b-5p augmented CRC progression by facilitating tumor growth and enhancing the aggressiveness of CRC cells [19]. Herein, our findings also highlighted that over-expression of miR-34b-5p suppressed the promoting effect of CAFs-EVs on CRC cell proliferation.

Subsequently, we predicted the target genes of miR-34b-5p, among which HuR was chosen as the subject of interest. Inherently, HuR, also known as ELAVL1, is a well-documented RNA-binding protein that exhibits notable tumorigenic activity [33]. Significant up-regulation of HuR has been previously documented in CRC tissues, which highlights the oncogenic role of HuR in CRC [34]. Our findings further demonstrated that HuR expression levels were elevated in CRC cells, whereas treatment with CAFs-EVs or CAFs-EVs-SNHG3 further up-regulated HuR expression, while over-expression of miR-34b-5p brought about the opposite trends. Additionally, HuR is also capable of binding UTRs of downstream mRNAs to stabilize the target mRNAs or enhance their translation [35]. Moreover, numerous mRNAs regulated by HuR encode certain proteins associated with progression of human malignancies [24]. For instance, the HOXC6 protein modulates cell differentiation during embryonic development, while aberrantly elevated HOXC6 expression levels are correlated with tumor progression [36]. Herein, our findings confirmed the binding relationship between HuR and HOXC6. After HuR siRNA was transfected into HCT116 cells, there was a reduction in HOXC6 mRNA expression levels. Meanwhile, existing evidence suggests that HOXC6 expressions are elevated in CRC in comparison to normal mucosa, such that higher HOXC6 expression levels are correlated with poorer overall survival [26]. Consistently, our findings illustrated that HOXC6 expression levels were enhanced in CRC cells, whereas treatment with CAFs-EVs or CAFs-EVs-SNHG3 further enhanced HOXC6 mRNA expression, while over-expression of miR-34b-5p led to a reduction in HOXC6 mRNA expression. Overall, our discoveries established that miR-34b-5p targeted HuR expression, thereby suppressing the binding of HuR to HOXC6 and reducing the stability of HOXC6 mRNA. Meanwhile, a prior study indicated that HOXC6 triggers CRC tumorigenesis through autophagy, whereas HOXC6 knockdown depresses CRC cell proliferation and attenuates autophagy [25]. Similarly, our findings exhibited that HOXC6 silencing depressed the promotive effect of CAFs-EVs on CRC cell proliferation. Furthermore, we established a murine xenograft model to validate the mechanism of CAFs-EVs in CRC proliferation in vivo. The subsequent results verified that CAFs-EVs carrying SNHG3 enhanced CRC cell proliferation in vivo *via* the miR-34b-5p/HuR/HOXC6 axis.

To sum up, our findings indicated that CAFs-EVs carrying SNHG3 enhanced CRC cell proliferation by competitively binding to miR-34b-5p, thereby reducing the binding of miR-34b-5p and HuR, elevating HuR expression, promoting the binding of HuR and HOXC6, and ultimately enhancing HOXC6 transcription. Nevertheless, there are a few limitations to our study. First, we solely explored one of the lncRNA carried by CAFs-EVs, while the role and mechanism of other lncRNAs remains to be explored. In addition, various other miRNAs downstream of lncRNA SNHG3 are also poorly expressed in the course of CRC. Whether these miRNAs are implicated in SNHG3 regulating CRC proliferation needs further exploration. In addition, we only determined the effect of CAFs-EVs on CRC cell proliferation, and its potential effects on other functions of CRC cells, such as migration and EMT, still remains elusive. We shall validate the effects of CAFs-EVs on other functions of CRC cells and explore more potential mechanisms of SNHG3 in our future endeavors.

## Materials and methods

### Ethics statement

The current study was conducted with the approval of the Ethical Committee of The Fourth Clinical Medical School of Guangzhou University of Chinese Medicine; Shenzhen Traditional Chinese Medicine Hospital. Signed informed consents were obtained from all participants prior to specimen collection. Animal experiments were carried out in accordance with the *Guide for the Care and Use of Laboratory Animals* [37]. Extensive efforts were made to minimize the number and suffering of the included animals. The reporting of this study conforms to ARRIVE 2.0 guidelines [38].

### Separation of human normal fibroblasts (NFs) and CAFs

Firstly, CAFs and NFs were separated from fresh CRC tissues and adjacent normal tissues (obtained >10 cm from the edge of tumor invasion) from 10 CRC patients (aged 55–76 years, including six males and four females) who did not undergo radiotherapy or chemotherapy prior to the operation. Natural fibroblasts were isolated within 1 h after tissue collection. Subsequently, the obtained tissues were washed with 75% alcohol and D-Hanks buffer to eliminate residual plasma. Next, the tissue sample was cut into small pieces using a sterile scalpel, and then detached with collagenase (1 mg/mL, Gibco, Grand Island, NY, USA) and hyaluronidase (0.5 mg/mL, Sigma-Aldrich, Merck KGaA, Darmstadt, Germany) at 37 °C for 1 h. The detached mixture was then centrifuged (1000 g, 5 min) and rinsed with phosphate-buffered saline (PBS) twice in DMEM (Gibco) to remove the fat and tissue fragments. Afterwards, the tissues were maintained in DMEM containing 15% fetal bovine serum (FBS) (Gibco) for 2 days. Following the removal of the suspended cells and tissues, fibroblasts, macrophages, and epithelial cells were the most adherent cells. After 3–5 days of culture, the macrophages and epithelial cells were found to be apoptotic, while only fibroblasts were preserved. The medium was refreshed every 2–3 days. Primary fibroblasts extracted from tumor tissues were regarded as CAFs, and primary fibroblasts extracted from normal tissues paired with tumor tissues were regarded as NFs. The aforementioned cells were maintained in DMEM comprising of 10% FBS and 1% penicillin-streptomycin (Gibco). The cells at passage 3 were used for subsequent experimentation.

### Identification of NFs and CAFs

Firstly, the prepared CAFs and NFs were observed under a microscope. Subsequently, the CAFs and NFs were seeded into a 24-well plate (at a density of 5000 cells/well), fixed with 4% paraformaldehyde (PFA) for 15 min, treated with 0.5% Triton-X-100 for 20 min, and blocked with 3% bovine serum albumin (BSA) for 1 h. Next, the cells were subjected to incubation with antibodies α-SMA (ab232784, Abcam, Cambridge, MA, USA), FAP (PA5–99313, Thermo Fisher Scientific, Waltham, MA, USA), or vimentin (ab92547, Abcam) at 4 °C overnight. The following day, the cells were incubated with the secondary antibody goat anti-rabbit IgG (ab6721, Abcam) for 45 min in conditions devoid of light. After removing the secondary antibody solution, the cells were rinsed with PBS thrice (5 min), incubated with 2,4-diaminobutyric acid (DAB) in dark conditions for 5 min, rinsed with PBS (2 × 5 min), counterstained with hematoxylin for 1 min, and sealed with neutral gum. Afterwards, the cells were observed under an optical microscope (CKX51, Olympus, Tokyo, Japan). Finally, the expression patterns of α-SMA, FAP, and vimentin were determined using reverse transcription quantitative polymerase chain reaction (RT-qPCR).

### Treatment of CAFs

To obtain a cell line stably-expressing SNHG3, we entrusted the Genechem company to amplify the cDNA of SNHG3 and sub-clone it into the lentivirus vector (GV248, with a puromycin selection gene, Genechem, Shanghai, China). Meanwhile, the lentivirus vector overexpressing lncRNA SNHG3 and its corresponding control lentivirus vector (NC) were synthesized and assembled by Genechem. CAFs stably overexpressing SNHG3 were selected by adding puromycin (2 μg/mL). In general, the isolated CAFs were seeded into 6-well plates, at a density of 2 × 10^5^ cells/well. Upon reaching 50%–60% confluence, lentivirus infection was carried out. Briefly, polybrene was diluted to a concentration of 50 μg/mL, and lentivirus was transfected with the multiplicity of infection of 50. Subsequently, the lentivirus was mixed with culture medium and polybrene diluent, and then added into the 6-well plates. After 72 h, CAFs were collected and EVs were isolated, and regarded as CAFs-EVs-NC and CAFs-EVs-SNHG3, respectively. In addition, CAFs were treated with RNase A alone (Sigma-Aldrich, 2 mg/mL) or RNase A combined with Triton-X-100 (0.1%) for 20 min to determine the presence of SNHG3 in CAFs-EVs.

### Isolation and identification of CAFs-EVs and NFs-EVs

CAFs-EVs and NFs-EVs were isolated by means of the ultracentrifugation method. Subsequently, the CAFs and NFs were seeded into 6-well plates (at a density of 2 × 10^5^), respectively and cultured with EVs-depleted serum (FBS was prepared by centrifugation at 100000 g and 4 °C). After 72 h, the medium was centrifuged (400 g, 4 °C, 5 min) to remove the floating cells, followed by centrifugation (3000 g, 4 °C, 20 min) to eliminate the cell debris. Next, the supernatant filtered through a 0.22 μm filter and centrifuged (100000 g, 4 °C, 4 h) to collect the particles in the supernatant and resuspend them in PBS, followed by 60-min of centrifugation (100,000 g and 4 °C) and PBS resuspension, and finally stored at −80 °C. Afterwards, the protein concentration in the solution was evaluated using bicinchoninic acid (BCA) kits (Beyotime, Shanghai, China). The morphology and particle size of EVs were evaluated with the help of a transmission electron microscope (TEM) (Hitachi, Tokyo, Japan) and Nanoparticle tracking analyzer (NTA) (Malvin Instrument Co., Ltd, Malvern, UK). The protein markers of EVs (CD9 and CD63) and negative control calnexin were detected using Western blot assay. The conditioned medium of CAFs added with GW4869 (10 μM; Sigma-Aldrich), the living EVs inhibitor, was regarded as the GW group.

### Cell culture

Human CRC cell lines HCT116 (derived from colon) and SW480 (derived from colon), and human normal colonic epithelial cell line NCM460 (derived from colon; mucosa) were supplied by American Type Culture Collection (Manassas, Virginia, USA). All cells received Short Tandem Repeat (STR) authentication. Then the cells were cultured with RPMI-1640 medium (Gibco) comprising of 10% FBS (Gibco), 100 U/mL penicillin, and 100 μg/mL streptomycin (Sigma-Aldrich) at 37 °C in a humidified incubator containing 5% CO_2_.

### Cell treatment

HCT116 or SW480 cells were seeded into 6-well plates and treated with 100 μg (50 μL PBS) NFs-EVs or CAFs-EVs, with equal amounts of NFs-GW or CAFs-GW treatment as NC. Afterwards, miR-34b-5p mimic or mimic NC (GenePharma, Shanghai, China), two Hu antigen R (HuR) siRNAs, and three homeobox C6 (HOXC6) siRNAs (RiboBio Co., Ltd, Guangzhou, China) were transfected into CRC cells, respectively using the Lipofectamine 3000 reagent (Invitrogen, Carlsbad, CA, USA). Subsequent experiments were carried out after 24 h.

### Co-culture of cy3-labeled SNHG3-transfected CAFs and CRC cells

To order to identify the transfer of CAFs-EVs lncRNA SNHG3, cy3-labeled SNHG3 (GenePharma, Shanghai, China) was transfected into CAFs and then co-cultured with HCT116 or SW480 cells in a 24-well Transwell chamber for 48 h. Afterwards, fluorescein isothiocyanate (FITC) phalloidin (Yeasen, Shanghai, China) was adopted to selectively stain the cytoskeleton of CRC cells. In addition, GW4869 was added to the above system as the EVs inhibition group. Finally, the internalization of CAFs-EVs lncRNA SNHG3 was observed by means of confocal microscopy (Leica Microsystems, Mannheim, Germany).

### Cell counting kit-8 (CCK-8) assay

Cell proliferation ability was evaluated with the help of CCK-8 assay kits (Dojindo, Kumamoto, Japan). HCT116 or SW480 cells were seeded into 96-well plates (at a density of 2 × 10^3^ cells/well), and cultured in humidified incubator at 37 °C with 5% CO_2_. Each well was added with 10 μL CCK-8 reagent every 24 h for another 12-h incubation at 37 °C. Afterwards, absorbance at 450 nm was assessed using a microplate reader (Bio-Rad, Philadelphia, PA, USA).

### Colony formation assay

HCT116 or SW480 cells were seeded in 6-well plates (at a density of 500 cells/well) and cultured at 37 °C for 14 days. After two PBS rinses, the cells were fixed with 4% PFA (700 μL per well, Sigma-Aldrich) for 20 min. Following another two PBS rinses, the cells were stained with 1% crystal violet (700 μL per well, Sigma-Aldrich), allowed to stand at room temperature for 30 min, rinsed with PBS, and finally, air-dried. Afterwards, the cell colonies were observed and photographed under an optical microscope (Olympus). NIH Image J (National Institutes of Health, Bethesda, Maryland, USA) was used for cell colony counting.

### 5-ethynyl-2'-deoxyuridine (EdU) staining

HCT116 or SW480 cells were detached with PBS in 12-well plates (at a density of 2 × 10^4^ cells/well) and resuspended. After 24 h, the cells were supplemented with 1% EdU solution (Beyotime) for 2-h of incubation at 37 °C. After the solution was removed, the cells were subsequently fixed with 4% PFA for 30 min, permeabilized with 0.3% Triton-X-100 solution for 15 min, and incubated with click workworn liquid in conditions devoid of light for 30 min. Finally, the membrane was sealed with a mounting medium containing 4,6-diamino-2-phenylindole (DAPI) (ab104139, Abcam), followed by photography under a fluorescence microscope (Olympus). The cell proliferation rate was expressed as the proportion of nucleated cells containing EdU ((EdU/DAPI) × 100%).

### Bioinformatics analysis

The subcellular localization of lncRNA SNHG3 was predicted through the lncLocator database (http://www.csbio.sjtu.edu.cn/bioinf/lncLocator/?tdsourcetag=s_pcqq_aiomsg) [39]. The downstream miRNAs of SNHG3 were predicted using the Starbase database (http://starbase.sysu.edu.cn/index.php) [40] and DIANA database (http://carolina.imis.athena-innovation.gr/diana_tools/web/index.php?r=lncbasev2%2Findex-predicted) [41]. The downstream genes of miR-34b-5p were predicted through Starbase, Targetscan (http://www.targetscan.org/vert_71/) [42], miRWalk (http://mirwalk.umm.uni-heidelberg.de/) [43], and miRDB databases (http://mirdb.org/index.html) [44]. In addition, the binding of HuR and HOXC6 was analyzed according to the RNA-Protein Interaction Prediction (RPISeq) database (http://pridb.gdcb.iastate.edu/RPISeq/) [45]. All designs were the default settings of the website and all analysis operations were carried out under the guidelines of the website.

### RNA fluorescence in situ hybridization (FISH)

FAM-labeled SNHG3 FISH probes were synthesized by RiboBio (Guangzhou, China). HCT116 or SW480 cells were placed on culture slides, fixed with 4% PFA (Sigma-Aldrich), and then blocked with pre-hybridization buffer for 4 h. Next, FISH probes were added to the hybridization mixture and incubated overnight. The following day, the slides were washed with washing buffer containing sodium citrate and stained with DAPI, followed by observation under a fluorescence microscope (Olympus).

### Nuclear/cytosol fractionation assay

Cytoplasmic and nuclear RNA content was extracted from HCT116 or SW480 cells using Cytoplasmic & Nuclear RNA Purification kit (Norgen Biotek, Canada) [24]. LncRNA SNHG3 expression patterns were determined using RT-qPCR, with GAPDH/U6 serving as the cytoplasmic/nuclear control.

### Dual-luciferase assay

The possible binding site of miR-34b-5p with SNHG3 or HOXC6 was obtained from the Starbase database. Next, the sequence of SNHG3 or HOXC6 containing wild-type (WT) or mutant-type (MUT) miR-34b-5p binding site was synthesized (RiboBio) and cloned into the pGL3 reporter gene vector (Promega Corporation, Madison, Wisconsin, USA). The vectors were co-transfected with miR-34b-5p mimic or mimic NC (GenePharma) into HCT116 or SW480 cells using the Lipofectamine 3000 reagent (Invitrogen) and incubated for 48 h. Afterwards, luciferase activity was measured using the Dual-Glo® Luciferase Analysis System (Promega).

### RNA pull-down

The biotin-labeled miR-34b-5p probe (Bio-miR-34b-5p) or negative control (Bio-miR-NC) was synthesized by GenePharma. Subsequently, the probe was transfected into HCT116 or SW480 cells. Next, the cells were harvested and lysed 48 h later. The biotinylated miR-34b-5p was captured using streptavidin magnetic beads and incubated with cell lysate at 4 °C overnight. Afterwards, the mixture was washed and eluted, and RNA enrichment was evaluated by means of RT-qPCR [46].

### RNA immunoprecipitation (RIP)

RIP assay was performed with the help of Magna RIP RNA-binding Protein Immunoprecipitation kits (Millipore, Billerica, MA, USA). Briefly, a total of 10^7^ HCT116 or SW480 cells were collected following trypsin treatment, lysed with 1 mL RIP buffer, incubated on ice for 10 min, and centrifuged (16000 g, 4 °C, 15 min). Subsequently, HuR (ab200342, Abcam) or IgG (ab172730, Abcam) antibodies were incubated with 1 mL cell lysate at 4 °C overnight. After incubation, 50 μL precleared protein A/G beads (Bimake, Houston, TX, USA) were added to the above compound and incubated at 4 °C for 4 h. Next, the magnetic beads were collected and washed thrice with 500 μL RIP buffer. Afterwards, the beads were resuspended in 1 mL TRIzol reagent for RNA extraction. HOXC6 mRNA expression patterns in immunoprecipitation samples and input were analyzed by means of RT-qPCR. RNA expression in immunoprecipitation samples was normalized to input.

### RNA stability analysis

HCT116 or SW480 cells were seeded into 12-well plates, at a density of 2 × 10^4^ cells/well. Following overnight incubation, the cells were treated with 10 μg/mL actinomycin D at different time intervals (0, 4, 8, and 12 h). Subsequently, the RNA content was extracted using the TRIzol reagent. The changes in RNA expressions were analyzed by means of RT-qPCR.

### Establishment of xenograft tumor model in nude mice

Male BALB/c mice (aged 6–8-week-old; weighing 18–20 g; procured from Vital River Laboratory Animal Technology Co., Ltd, Beijing, China) [SYXK (Beijing) 2017–0033] were placed in a standard animal room and maintained under a 12-h light/dark cycle, with *ad libitum* access to food and water. The mice were numbered according to their body weight, and then, the nude mice were randomly grouped using the random number method and recorded by the experimenters. Prior to the animal experiment, as described in previous literature [10], a total of 1.5 × 10^6^ HCT116 cells were cultured in medium and treated with different concentrations of CAFs-EVs (100 μg/50 μL PBS), with equal amounts of CAFs-GW treatment as the negative control and untreated cells as the Control group. After 12 h, HCT116 cells were rinsed with PBS to eliminate excess EVs and prepare cell suspension. According to different treatments, the mice were allocated into the following 5 groups (12 mice in each group): the control group, the CAFs-GW group, the CAFs-EVs group, the CAFs-EVs-NC group, and the CAFs-EVs-SNHG3 group. The prepared cell suspension (2 × 10^6^ cells/200 μL) was subcutaneously injected into the right groin area of nude mice. The length (L) and width (W) of the tumor were measured using vernier calipers every 7 days to monitor the growth of the tumor. The tumor volume (V) was calculated using the following formula: *V* = 0.5 × *L* × *W*^2^. The health and behavior of all animals were detected every 2 days. The nude mice were euthanized when the following conditions (humanitarian end point) occurred: weight loss >10% of the weight of nude mice, or the animals suffered from tumor burden, or the maximum diameter of the tumor exceeded 1.5 cm. There was no midway death of animals in the experiment. Afterwards, the mice were euthanized with an intraperitoneal injection of ≥100 mg/kg pentobarbital sodium on the 28th day after the operation. Tumor resection was performed and tumor weight was recorded. In order to minimize the number of mice and ensure the number of repetitions of experimental data and the reliability of sample size, we collected tumor specimens from six mice in each group for immunohistochemical staining to detect the expression patterns of Ki67 and HuR, and extracted tumor specimens from the remaining six mice in each group for subsequent RT-qPCR.

### Immunohistochemistry

The tumor tissues were fixed with 4% PFA, paraffin-embedded, and sectioned (4 μm). Following dewaxing and rehydration, the sections were treated with H_2_O_2_ for 8 min to eliminate endogenous peroxidase activity, and then blocked with 3% BSA for 1 h. The sections were incubated with anti-Ki67 (ab15580, Abcam) or HuR (ab200342, Abcam) overnight. Following three PBS rinses, the sections were subjected to 1-h incubation with the secondary antibody (ab6721, Abcam) and 5-min reaction with DAB solution (Yeasen). Finally, the sections were counterstained with hematoxylin (Beyotime) for 1 min and sealed with neutral gum after dehydration, followed by observation under a microscope (Olympus CKX51). Five visual fields were selected from each section to count the number of positive cells [47]. A double-blind analysis was conducted by two experts who were completely unaware of the experiment and research.

### RT-qPCR

Total RNA content was isolated from cells, EVs, or tumor tissues using the TRIzol reagent (Invitrogen) and then reverse-transcribed into cDNA with PrimeScript RT Master Mix (TaKaRa, Dalian, China). RT-qPCR was subsequently conducted using SYBR Premix Ex Taq (TaKaRa) as follows: pre-denaturation at 95 °C for 10 min, and 40 cycles of denaturation at 95 °C for 30 s, annealing at 55 °C for 30 s, and extension at 72 °C for 30 s. The ABI Prism 7500 Sequence Detection System (Applied Biosystems, Foster City, CA, USA) was adopted for data collection. Supplementary Table 1 shows PCR primers. The relative expression of genes was calculated using the 2^−ΔΔCt^ method [48], with GAPDH and U6 serving as the internal references [21].

### Western blotting

Total protein content of EVs or cells was extracted using radio-immunoprecipitation assay buffer containing 1% protease inhibitor PMSF, and the protein concentration was determined with the help of BCA assay kits (Beyotime). The obtained proteins were separated using 10% SDS-PAGE and transferred onto PVDF membranes (Millipore). Subsequently, the membranes were blocked with 5% 5% milk-Tris-buffer saline containing 0.1% Tween-20 (TBST) at 37 °C for 1 h. Next, the membranes were incubated with the following primary antibodies: HuR (ab200342, dilution ratio of 1:1000, Abcam), CD63 (ab134045, dilution ratio of 1:1000, Abcam), CD9 (ab92726, dilution ratio of 1:2000, Abcam), Calnexin (ab92573, dilution ratio of 1:20000, Abcam), and β-actin (ab8227, dilution ratio of 1:1000, Abcam) at 4 °C overnight. The following day, after TBST washing, the membranes were subjected to 1-h incubation with the secondary antibody (ab6721, dilution ratio of 1:2000, Abcam) at 37 °C. Afterwards, the gray values were analyzed using the NIH Image J software.

### Statistical analysis

Data analysis and map plotting were carried out using the SPSS 21.0 (IBM Corp., Armonk, NY, USA) and GraphPad Prism 8.0 software (GraphPad Software Inc., San Diego, CA, USA), respectively. Measurement data are expressed as mean ± standard deviation. Comparisons between two groups were performed using the *t*-test, while comparisons among multiple groups were carried out using one-way or two-way analysis of variance (ANOVA), followed by Tukey’s multiple comparison test. A value of *p* < 0.05 was considered statistically significant.

## Supplementary information

Supplementary figure 1

Supplementary figure 2

Supplementary Figure 3

Supplementary Figure 4

Supplementary Figure 5

Supplementary Figure Legends

Supplementary Table 1

## Data Availability

The data that support this study are available from the corresponding author upon reasonable request.
