# Supplementary figures and images for: Cancer-associated fibroblasts-derived extracellular vesicles carrying lncRNA SNHG3 facilitate colorectal cancer cell proliferation via the miR-34b-5p/HuR/HOXC6 axis
Source: Cell Death Discov. 2022 Aug 3;8:346. doi: 10.1038/s41420-022-01116-z (PMC9349187; doi:10.1038/s41420-022-01116-z)

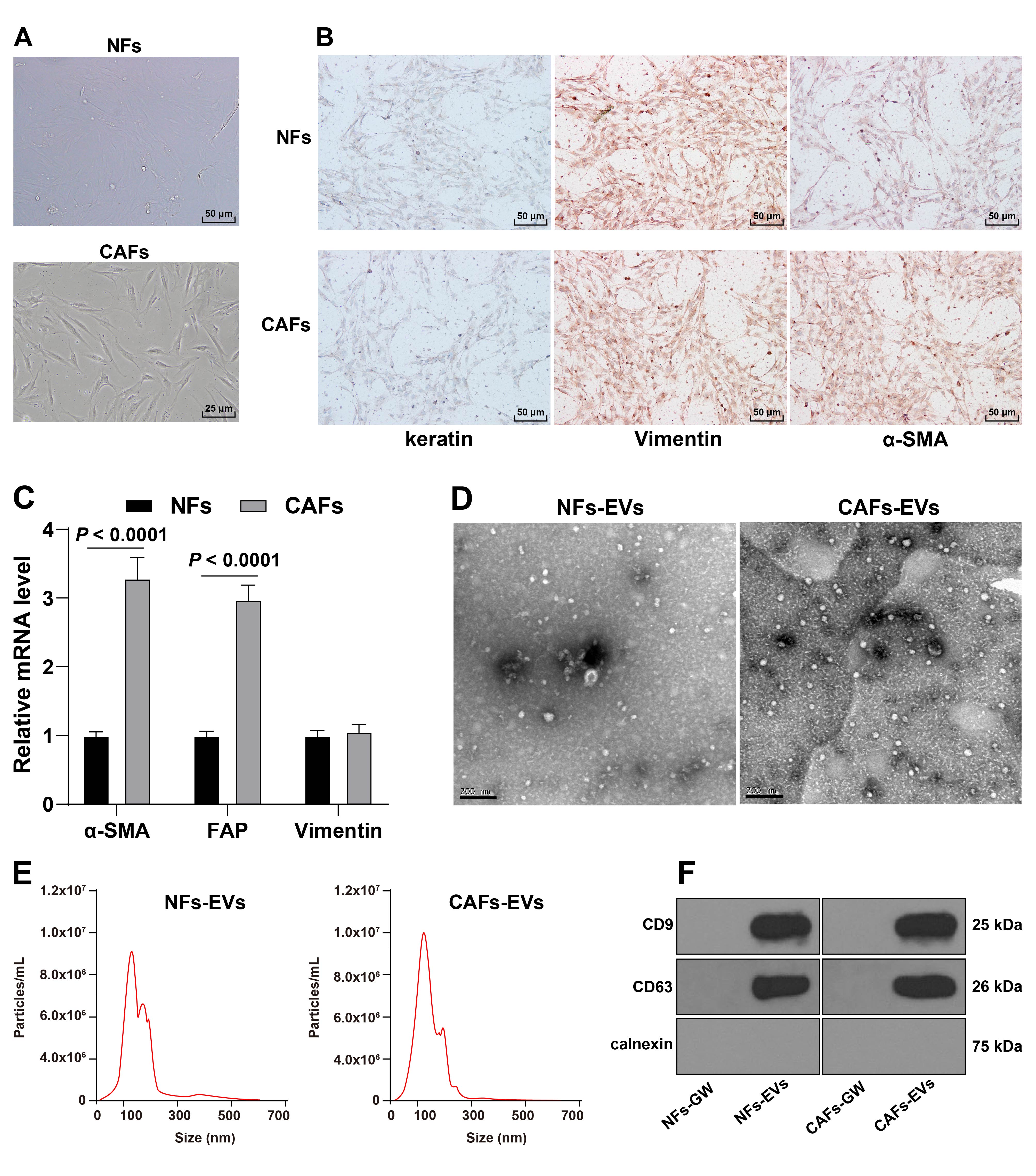

Supplement: Supplementary file 1 — Supplementary figure 1 [file 41420_2022_1116_MOESM1_ESM.jpg]

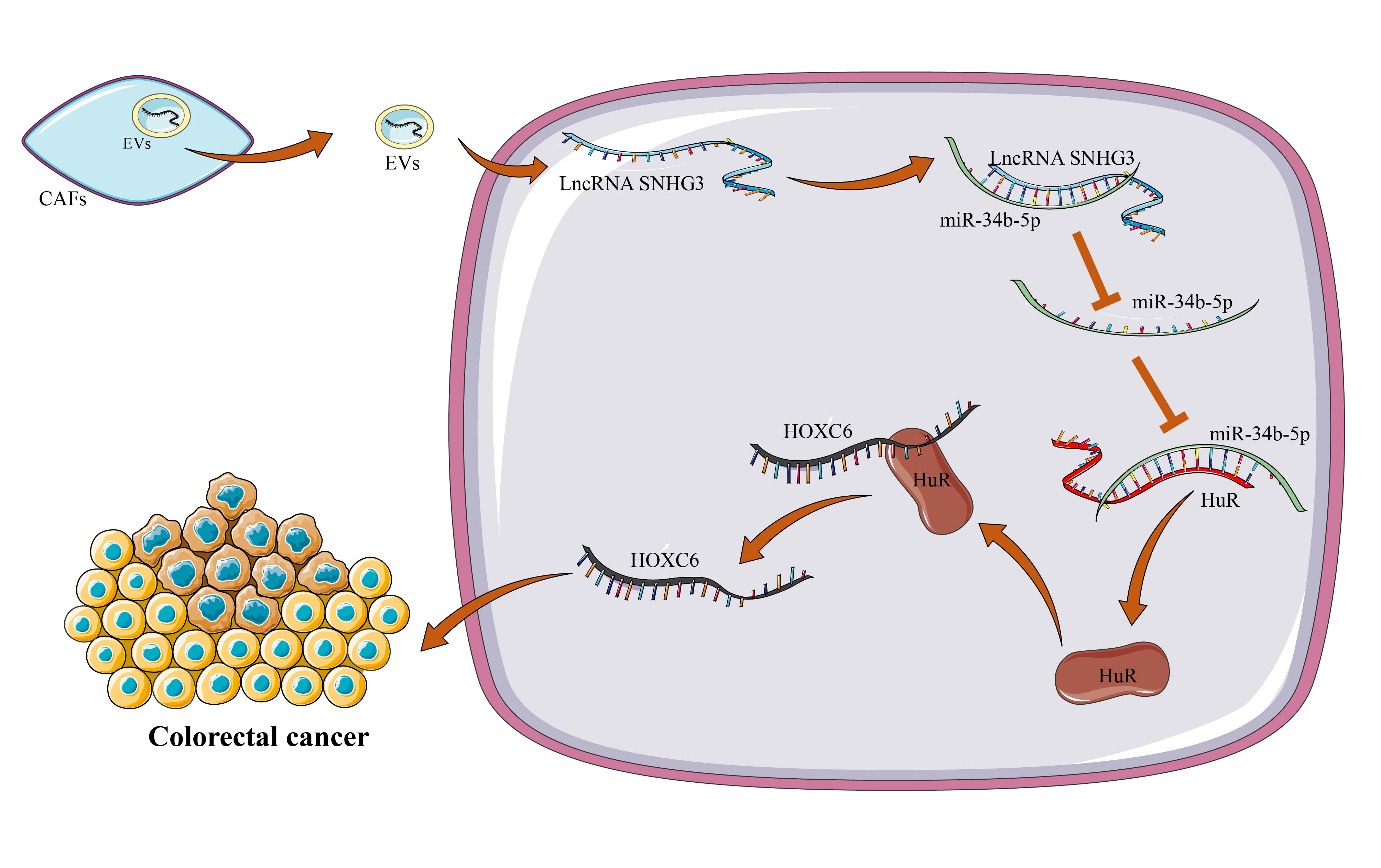

Supplement: Supplementary file 2 — Supplementary figure 2 [file 41420_2022_1116_MOESM2_ESM.jpg]

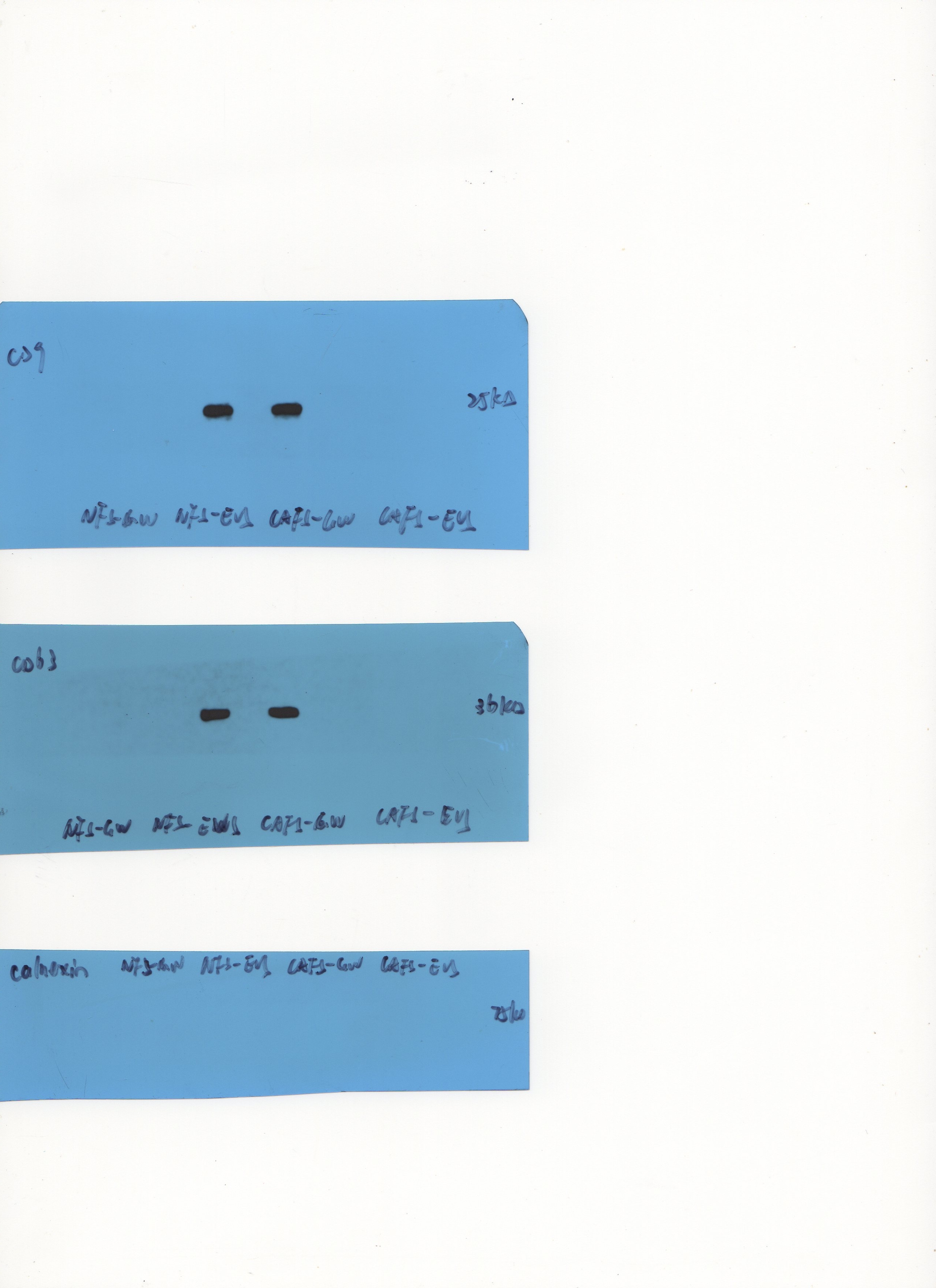

Supplement: Supplementary file 3 — Supplementary Figure 3 [file 41420_2022_1116_MOESM3_ESM.jpg]

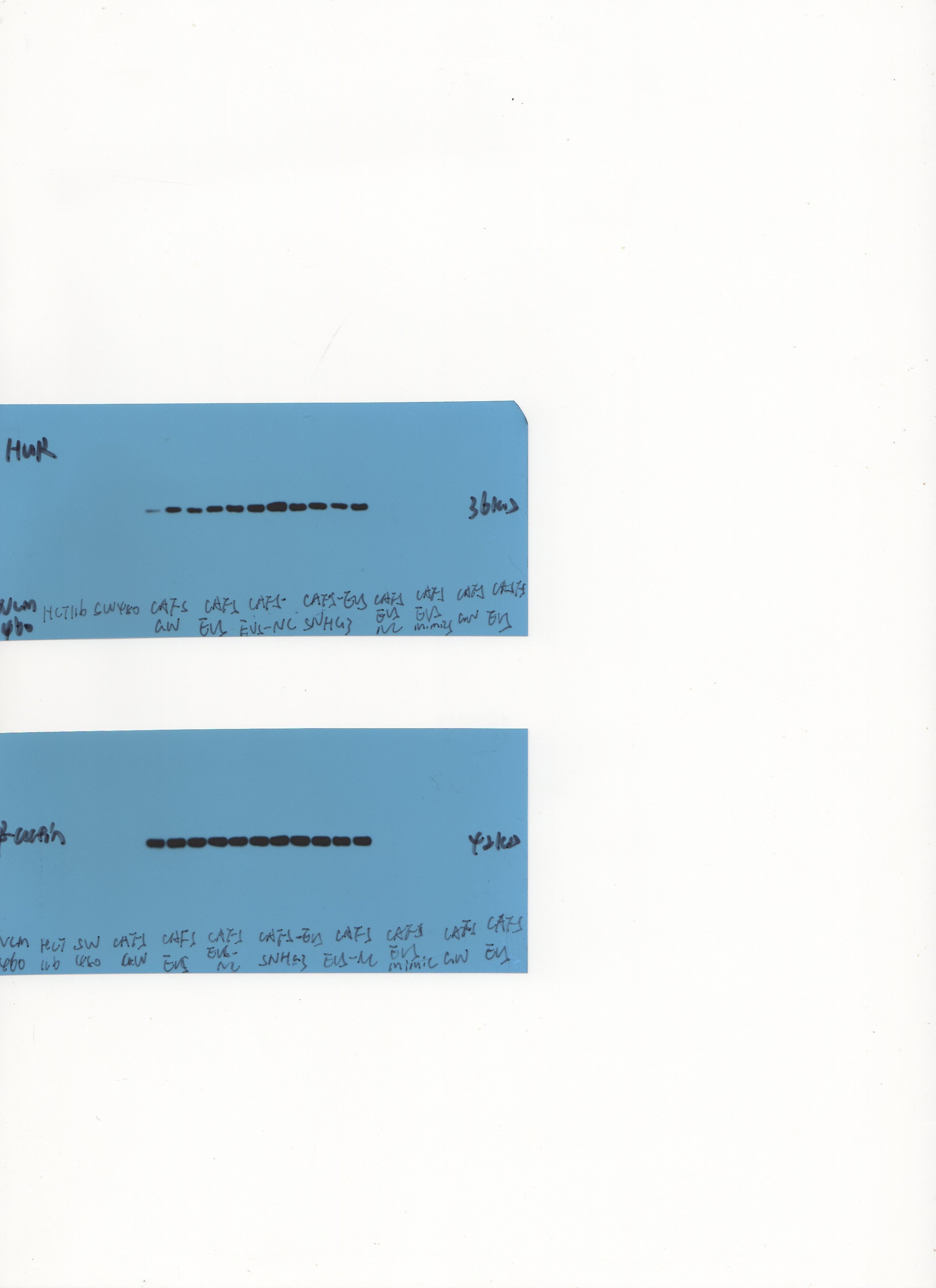

Supplement: Supplementary file 4 — Supplementary Figure 4 [file 41420_2022_1116_MOESM4_ESM.jpg]

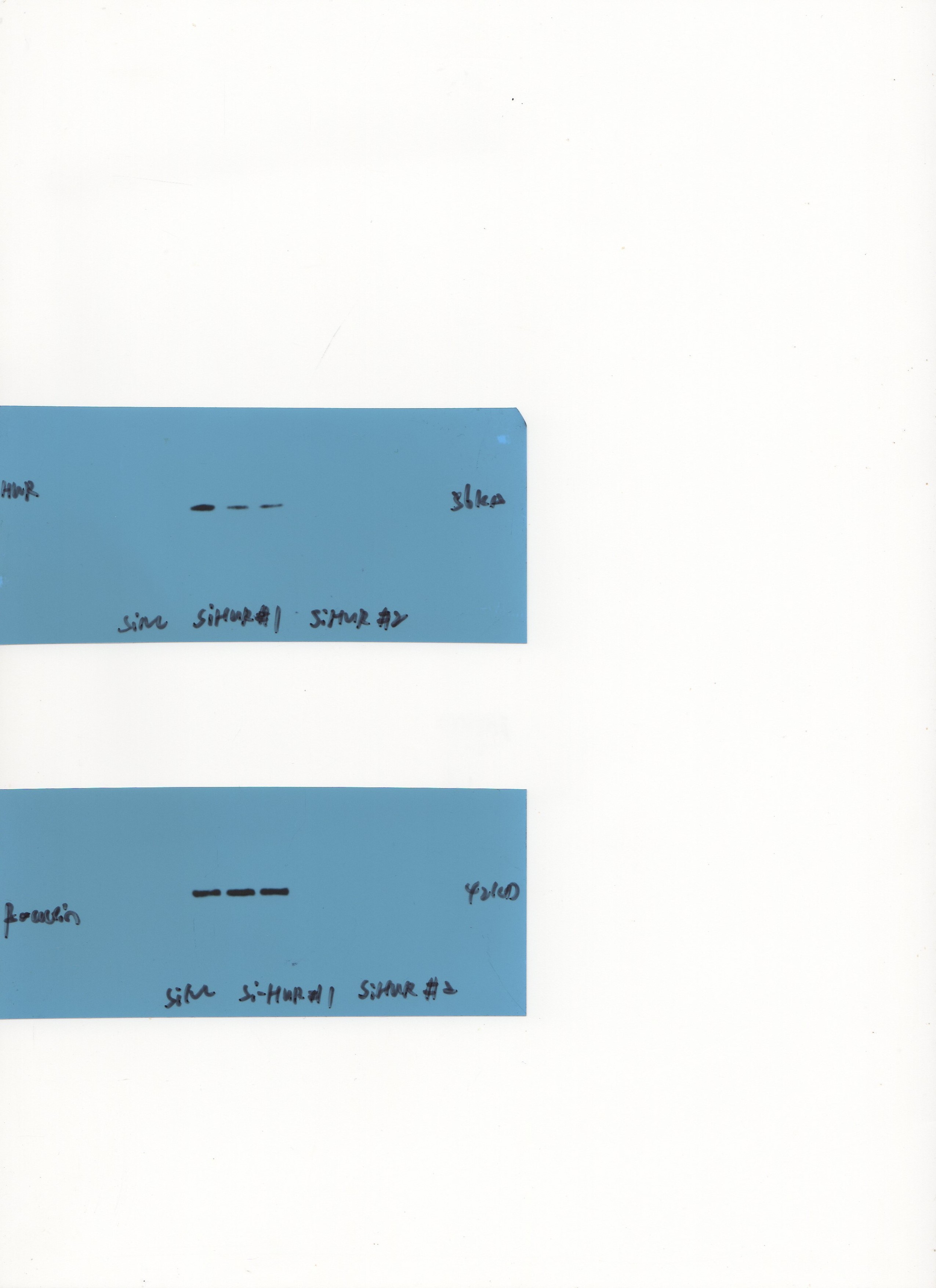

Supplement: Supplementary file 5 — Supplementary Figure 5 [file 41420_2022_1116_MOESM5_ESM.jpg]
