# Supplementary material for: Cancer-associated fibroblasts-derived extracellular vesicles carrying lncRNA SNHG3 facilitate colorectal cancer cell proliferation via the miR-34b-5p/HuR/HOXC6 axis
Source: Cell Death Discov. 2022 Aug 3;8:346. doi: 10.1038/s41420-022-01116-z (PMC9349187; doi:10.1038/s41420-022-01116-z)
Supplement: Supplementary file 6 — Supplementary Figure Legends [file 41420_2022_1116_MOESM6_ESM.docx]

**Supplementary Figure Legends**

**Supplementary Figure 1** Isolation and characterization of NFs, CAFs, and their EVs. Human NFs, CAFs, and their EVs were isolated. A: The morphology of NFs and CAFs was observed under the microscope. B: The expressions of specific markers (keratin, vimentin, and α-SMA) were detected using immunocytochemistry. C: The mRNA expressions of specific markers (α-SMA, FAP, and vimentin) were detected using RT-qPCR. D: The morphology of NFs-EVs and CAFs-EVs was observed under the transmission electron microscope. E: The concentration and particle size of NFs-EVs and CAFs-EVs were measured using a nanoparticle tracking analyzer. F: The expressions of surface markers of NFs-EVs and CAFs-EVs (CD9, CD63, and calnexin) were determined using Western blotting. The cell experiment was repeated 3 times independently. Data are presented as mean ± standard deviation. Data in panel C were analyzed using two-way ANOVA, followed by Tukey's multiple comparisons test.

**Supplementary Figure 2** Mechanism of CAFs-EVs carrying SNHG3 in the promotion of CRC cell proliferation. CAFs-EVs carried SNHG3 into CRC cells to promote the binding of SNHG3 and miR-34b-5p and then reduce miR-34b-5p expression, thereby inhibiting the binding of miR-34b-5p and HuR, upregulating HuR expression and promoting the binding of HuR and HOXC6, increasing HOXC6 RNA stability and enhancing HOXC6 transcription, and eventually facilitating CRC cell proliferation.

**Supplementary Figure 3** Additional data of full and uncropped western blots. The expressions of surface markers of NFs-EVs and CAFs-EVs (CD9, CD63, and calnexin) were determined using western blotting.

**Supplementary Figure 4** Additional data of full and uncropped western blots. HuR expression in NCM460 and CRC cells was determined using western blotting.

**Supplementary Figure 5** Additional data of full and uncropped western blots. HuR expression in HCT116 cells was determined using western blotting.
