# Supplementary material for: Cancer-associated fibroblasts-derived extracellular vesicles carrying lncRNA SNHG3 facilitate colorectal cancer cell proliferation via the miR-34b-5p/HuR/HOXC6 axis
Source: Cell Death Discov. 2022 Aug 3;8:346. doi: 10.1038/s41420-022-01116-z (PMC9349187; doi:10.1038/s41420-022-01116-z)
Supplement: Supplementary file 7 — Supplementary Table 1 [file 41420_2022_1116_MOESM7_ESM.docx]

**Supplementary Table 1** Primer sequence for RT-qPCR

| Name | Sequence (5’-3’) |
| --- | --- |
| LncRNA SNHG3 | F: CTGCACTTCGCATTTTGGCA |
|  | R: TGCACCTCAATCTTTTGCTCC |
| miR-34b-5p | F: GCATGCTAGGCAGTGTCATTAG |
|  | R: CTCAACTGGTGTCGTGGAGTC |
| ELAVL1 (HuR) | F: TCCGGCTGGTGCATTTTCAT |
|  | R: AGTCGCGGATCACTTTCACA |
| HOXC6 | F: TAACCCTTCCTTATCCTGCCAC |
|  | R: CTCACTGGATCATAGGCGGT |
| α-SMA | F: CATCACCAACTGGGACGACA |
|  | R: CAGGGTGGGATGCTCTTCAG |
| FAP | F: AGAACCATGCTTTGGAGATACT |
|  | R: TTTACTCCCAACAGGCGACC |
| vimentin | F: GTGGACCAGCTAACCAACGA |
|  | R: AGGGTGTTTTCGGCTTCCTC |
| GAPDH | F: ATGGTTTACATGTTCCAATATGA |
|  | R: TTACTCCTTGGAGGCCATGTGG |
| U6 | F: TCGCTTCGGCAGCACATATACT |
|  | R: GCTTCACGAATTTGCGTGTCATC |

Note: RT-qPCR: reverse transcription quantitative polymerase chain reaction; LncRNA SNHG3: Long noncoding RNA small nucleolar RNA host gene 3; miR-34b-5p: microRNA-34b-5p; ELAVL1: embryonic lethal abnormal vision 1; HuR: Hu antigen R; HOXC6: homeobox C6; FAP: fibroblast activation protein; GAPDH: glyceraldehyde-3-phosphate dehydrogenase
